# *Bacillus subtilis* Fed to Sows Promotes Intestinal Development and Regulates Mucosal Immunity in Offspring

**DOI:** 10.3390/vetsci12050489

**Published:** 2025-05-18

**Authors:** Peng Liu, Jinjiao Zuo, Hui Lu, Bin Zhang, Caihong Wu

**Affiliations:** 1 College of Veterinary Medicine, Jiangsu Agri-Animal Husbandry Vocational College, Taizhou 225300, China; jsmytg@126.com; 2 Pet Science and Technology College, Jiangsu Agri-Animal Husbandry Vocational College, Taizhou 225300, China; 19515812238@139.com (J.Z.); binzhang021@126.com (B.Z.)

**Keywords:** *Bacillus subtilis*, sow, piglet, intestinal development, mucosal immunity

## Abstract

Diarrhea in piglets causes intestinal inflammation and epithelial damage. Weaned piglets fed with *Bacillus subtilis* (B.S) have enhanced intestinal mucosal immunity and reduces diarrhea in piglets. However, the immune system of newborn piglets is immature, and B.S cannot effectively activate the intestinal mucosal reaction when given directly. This research explored the impact of the maternal supplementation of B.S-Dia during the final 35 days of gestation on piglet intestinal development and mucosal immunity. The results demonstrated that B.S-Dia administration significantly increased the body weight, jejunal villus height, and crypt depth in the piglets. In addition, B.S-Dia also significantly increased the proliferative activity of intestinal epithelial cells, as evidenced by proliferating cell nuclear antigen (PCNA) staining and the elevated mRNA expression of the proliferation-related gene (*c-Myc*). Furthermore, B.S-Dia supplementation also reinforced the intestinal mucosal barrier by increasing goblet cell numbers and upregulating the mRNA expression of antimicrobial peptides, such as *Muc2* and *Lyz-1*. Finally, elevated levels of IL-4 and IFN-γ, along with an increased abundance of CD3^+^ T cells, revealed that the intestinal mucosal immunity of piglets was improved after B.S-Dia administration. Our study indicates that feeding B.S-Dia to sow spromotes intestinal development and improves intestinal mucosal immunity in piglets.

## Simple Summary

Piglet diarrhea is a common problem in intensive farming systems and is mainly characterized by intestinal inflammation and epithelial damage, leading to high mortality among piglets. Therefore, the intestinal tract has a major influence on the early health status of newborn piglets. Numerous studies have revealed that feeding weaned piglets *Bacillus subtilis* improves their immune function and intestinal integrity, thereby reducing diarrhea-related mortality. However, because the mucosal immune system in the intestine of newborn piglets is not fully developed, the use of *Bacillus subtilis* to improve diarrhea is not ideal. In this study, we aim to evaluate the impact of dietary supplementation with recombinant *Bacillus subtilis* capable of producing 4,4′-diaponeurosporene (B.S-Dia) in sows at gestation day 80, focusing on its effects on intestinal development and mucosal immunity of newborn piglets. The results are expected to offer a theoretical foundation for using B.S-Dia to boost immune function in piglets.

## 1. Introduction

Piglet diarrhea is a common problem in intensive farming systems, mainly characterized by intestinal inflammation and epithelial damage and leading to high mortality among piglets [1]. Therefore, the intestinal tract has a major influence on the early health status of piglets. Its development is a complex, dynamic process that initiates before birth and continues throughout the postnatal period [2]. Due to the underdevelopment of the gastrointestinal system in newborn piglets, they are highly susceptible to external antigens, such as natural toxins, pathogens, and even certain commensal microbes [3]. Diarrhea caused by pathogens remains the most frequent and recurrent condition in piglets. Some studies have indicated high morbidity and mortality caused by enterovirus in piglets, such as transmissible gastroenteritis virus (TGEV), porcine epidemic diarrhea virus (PEDV), and rotavirus A (RVA), directly resulting in considerable economic losses [4,5].

Besides its role in digestion and the absorption of nutrients, the intestine acts as an important defense against various pathogens [6]. The intestinal mucosal barrier primarily consists of epithelial cells, mucus layers, and components of the mucosal immune system [7]. The epithelial cells are bound tightly together, thereby separating the contents from the intestinal tissues and maintaining gut health [8]. The destruction of the mucosal barrier of the intestine leads to a series of pathological changes, including increased epithelial permeability and damage to the intestinal architecture. In more severe cases, pathogens may be transferred to other viscera, for instance, the liver, resulting in whole-body pathological responses [9,10].

Specific antimicrobial peptides (AMPs), such as Lyz-1 (lysozyme 1) and Muc2 (mucin 2), play important roles in gut immune responses. Lyz-1, secreted by Paneth cells, prevents infection by hydrolyzing bacterial cell walls. Muc2, primarily secreted by goblet cells, is a key component of the mucus layer that protects the intestinal barrier and regulates microbial colonization. AMP gene expression in the normal pig intestine is regulated, with differential mRNA expressions of AMPs, such as *Lyz-1* and *Muc2*, in different parts of the gut. Studies have shown that the intestinal immune system upregulates the mRNA expression of these AMPs in response to pathogen invasion, maintaining the stability of the gut mucosal barrier [11,12]. To further investigate the effects of 4,4′-Diaponeurosporene-producing *Bacillus subtilis* (B.S-Dia) on the expression of these AMP genes, we chose these AMPs as important targets in our study.

*Bacillus subtilis* (B.S) is a frequently used probiotic that regulates the intestinal microflora, improves growth performance, and enhances immunity in animals [13,14]. The lipopolysaccharide produced after B.S lysis is recognized by the Toll-like receptor 4 (TLR4) on the surface of dendritic cells (DCs), thereby enhancing local mucosal immunity by promoting DC maturation [15]. Numerous studies revealed that feeding weaned piglets B.S improved their immune function and intestinal integrity, thereby reducing their mortality caused by diarrhea [16,17,18]. In newborn piglets, the intestinal mucosal immune system is immature, which limits the effectiveness of B.S supplementation in preventing diarrhea. Interestingly, several studies have reported that feeding B.S to sows can improve the growth performance of their offspring [19,20,21]. In this study, we hypothesized that maternal supplementation with B.S-Dia during the late gestation period would enhance intestinal development and mucosal immune function in piglets by promoting epithelial proliferation and strengthening the mucosal barrier. To our knowledge, this is one of the first studies to investigate the effects of B.S-Dia supplementation in sows and its influence on the health of their offspring. Our objective was to evaluate whether B.S-Dia administration during late pregnancy would improve intestinal morphology and mucosal immunity in piglets, which may offer strong support for the future utilization of B.S-Dia.

## 2. Materials and Methods

### 2.1. Animals

In this study, ten second-parity sows (Large White × Landrace crossbreed) were derived from the Hongsong piggery located in the Gaochun District of Jiangsu Province. A total of 112 healthy neonatal piglets (57 in the control group and 55 in the B.S-Dia group), with approximately equal numbers of males (*n* = 54) and females (*n* = 58), were born to ten sows (five per group). All sows and piglets were housed in environmentally controlled farrowing pens under standard commercial conditions, with the temperature maintained at 28–30 °C for sows and 32–34 °C for piglets during the first week after birth. The relative humidity was kept at approximately 60–70%, and adequate ventilation was ensured. The sows were fed a standard commercial lactation diet formulated to meet NRC (2012) [22] nutrient requirements, and the piglets were allowed to suckle ad libitum. Clean water was available at all times.

### 2.2. Experimental Design and Sample Collection

Ten sows selected at random from the swine farm were allocated to two groups: the B.S-Dia group and the phosphate buffered saline (PBS) group, to which 5 mL B.S-Dia (1 × 10^10^ CFU/mL) and PBS, respectively, was orally gavaged during the last 35 days of gestation (GD80) [23]. All sows received oral administration at 5-day intervals up to parturition (GD115) [23]. The piglets were allowed to freely suckle colostrum and milk from their dams until 8 days of age. The body weights of all piglets were recorded daily from day 1 to day 8 post-birth. The average litter weight was calculated and used for growth performance analysis. Then, one piglet per litter (a total of five piglets per group) was randomly selected and euthanized via pentobarbital sodium injection (100 mg/kg). The jejunum was carefully dissected from each piglet. A portion of the jejunal tissue was fixed in 4% paraformaldehyde for histological and immunohistochemical analyses, while another portion was gently flushed with cold PBS to remove the intestinal contents, snap-frozen in liquid nitrogen, and stored at −80 °C for subsequent RNA extraction and qPCR analysis.

### 2.3. Histological and Immunohistochemical Analysis

Jejunal specimens were preserved in 4% paraformaldehyde for 24 h and subsequently passed through sequential ethanol solutions of increasing concentration (75%, 85%, 95%, and 100%), cleared using xylene, and embedded in paraffin. Paraffin blocks were sectioned into 5 μm slices using an ultra-thin semiautomatic microtome and mounted onto glass slides. The slides were dried overnight in a 37 °C incubator. Before staining, the sections were deparaffinized in xylene and rehydrated through graded ethanol to distilled water.

For hematoxylin and eosin (H&E) analysis, the sections were rehydrated and then stained with HE, followed by dehydration, clearing, and mounting. For each section, five complete villus–crypt units were randomly selected and measured under different fields of view. Two sections were analyzed per piglet. The average of all measurements from each piglet was used for statistical analysis. Jejunal villus height and crypt depth were assessed via a blinded observer to ensure objectivity and accuracy.

For periodic acid-schiff (PAS) staining, rehydrated sections were stained with Schiff’s reagent for 15 min at room temperature (RT), rinsed with running tap water for 10 min, and counterstained with hematoxylin for 2 min. After a brief differentiation step (acid solution, 3 s), the sections were rinsed with water for 15 min, dehydrated, cleared, and mounted. Goblet cell numbers were counted under light microscopy. For quantification, the goblet cells were counted in five randomly selected villus units per section under a light microscope. Two sections were evaluated per piglet. The average number of goblet cells per villus unit from each piglet was used for statistical analysis.

For immunohistochemistry (IHC), antigen retrieval was performed in citric acid–sodium citrate buffer (pH 6.0) for 15 min at 95 °C. Endogenous peroxidase activity was blocked with 3% H_2_O_2_ at 37 °C for 1 h, followed by blocking with 5% bovine serum albumin for 1 h to reduce non-specific binding. The sections were incubated overnight at 4 °C with a primary antibody against proliferating cell nuclear antigen (PCNA, 1:100, Abcam, Cambridge, Cambridgeshire, UK), and subsequently incubated with a biotinylated secondary antibody and streptavidin–HRP complex from the SABC kit (Boster, Wuhang, Wuhan, China). Color development was achieved with 3,3’-diaminobenzidine (DAB), and the sections were counterstained, dehydrated, cleared, and mounted. For quantification, five crypt units per section were randomly selected to count PCNA-positive nuclei. Two sections were analyzed per piglet. The proliferation index was calculated as the percentage of PCNA-positive cells among the total cells in the crypts. The measurements were performed by a blinded observer.

### 2.4. Immunofluorescence

Tissue sections were dewaxed and subjected to antigen retrieval by heating in citric acid–sodium citrate buffer (pH 6.0) for 15 min. After rinsing with PBS, non-specific binding was blocked by incubating the sections with 5% bovine serum albumin for 1 h. Subsequently, the sections were incubated overnight at 4 °C with rabbit anti-pig CD3 primary antibodies (bs-10498R, Beijing Boasen Biotech Co., Ltd., Beijing, China) following five PBS washes. The following day, an APC-conjugated secondary antibody (bs-0309R-APC, Beijing Boasen Biotech Co., Ltd., Beijing, China) was applied and incubated for 2 h. After washing again with PBS, the cell nuclei were counterstained with 4′,6-Diamidino-2-phenylindole (DAPI, 564907, BD Biosciences, Franklin Lakes, NJ, USA). CD3^+^T cells in the intestinal tissue were visualized using fluorescence confocal microscopy.

### 2.5. ELISA

Before euthanasia, blood samples were taken from the piglets’ cranial vena cava. The samples were spun at 4 °C 5000 rpm for 5 min, and the serum was collected. Then, we randomly collected a section of the jejunum and injected 5 mL of precooled sterile PBS into the intestine to rinse it. This liquid was then fully mixed, collected, and centrifuging at 5000 rpm for 10 min to collect the supernatant. Finally, the SIgA, IL-4 and IFN-γ in the intestinal rinse were assessed using ELISA kits (Shanghai Huyu Biotechnology Co., Ltd., Shanghai, China) according to the manufacturer’s instructions.

### 2.6. Real-Time PCR Quantification

Total ribonucleic acid (RNA) was extracted from the jejunal tissue samples using TRIzol^®^ reagent (T9424, Merck KGaA, Darmstadt, Germany) according to the manufacturer’s instructions. RNA purity and concentration were determined using a UV spectrophotometer (840-317400, Thermo Scientific™), and then cDNA was obtained through reverse transcription according to the instructions of the HiScript TM QRT SuperMix kit (R222-01, Vazyme, Nanjing, Jiangsu, China). The cDNA was amplified with SYBR Green qPCR. The mRNA expression of the *c-Myc* (related to cell proliferation) and *Lyz-1* and *Muc2* (related to innate defense in the intestine) in piglets’ intestines were measured using relative quantitative PCR. Gene expression levels of *c-Myc*, *Lyz-1*, and *Muc2* were quantified using GAPDH as the internal control. The primer sequences are provided in Table S1. Relative gene expression was calculated using the 2^−ΔΔCt^ method, where ΔCt represents the difference in threshold cycle (Ct) values between the target and reference genes (GAPDH). All data were normalized to GAPDH expression to ensure reliable comparisons between groups.

### 2.7. Flow Cytometry

Lymphocytes were isolated from 10 mL of peripheral blood using density gradient centrifugation. Briefly, equal volumes of blood and lymphocyte separation solution (10 mL each) were layered in a 50 mL sterile centrifuge tube and centrifuged at 2000 rpm for 25 min. The intermediate buffer coat layer was carefully collected, washed twice with RPMI medium, and resuspended in 100 μL PBS. CD3^+^T cells were then identified via flow cytometry using anti-pig CD3ε antibody (561476, BD Biosciences, Franklin Lakes, NJ, USA). A minimum of 10,000 events within the lymphocyte gate (based on forward and side scatter) were collected per sample. Isotype-matched control antibodies (557714, BD Biosciences, Franklin Lakes, NJ, USA) were used to set gates and assess the specificity of staining.

### 2.8. Statistical Analysis

Data were tested for normality (Shapiro–Wilk test) and homogeneity of variance (Levene’s test) before statistical analysis. An unpaired two-tailed Student’s *t*-test was used to compare differences between the two groups. Data are presented as the mean ± standard deviation (SD), and significance was set at *p* < 0.05. All statistical analyses were performed using GraphPad Prism 9. Blinding was applied during data analysis, which was conducted by an investigator unaware of the treatment assignments.

## 3. Results

### 3.1. Effects of Feeding B.S-Dia to Sows on the Weight and Intestinal Morphology of Piglets

**Figure 1:**
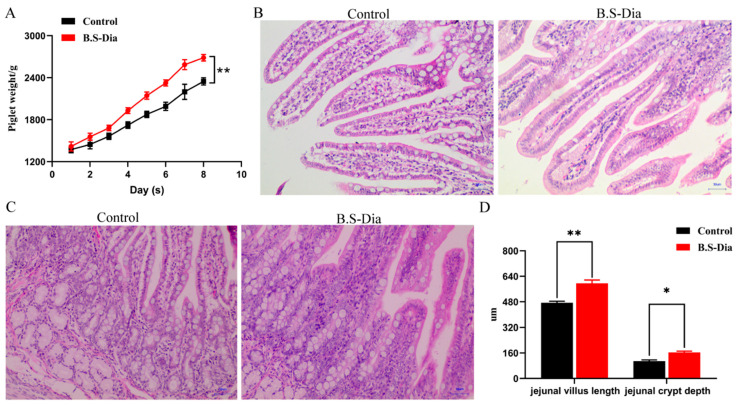
Impact of B.S-Dia treatment on weight gain and jejunal histology of piglets. (**A**) The mean weight gain of piglets per litter for the two groups (measured in grams). (**B**,**C**) Hematoxylin-eosin staining of jejunal villi and crypts in samples from PBS and B.S-Dia groups. Original magnification ×100, ×400, scale bar = 50 μm. (**D**) Systematic analysis of jejunal villus length and crypt depth in piglets. Data are shown as the mean ± S.D, and the significant difference was determined using a *t*-test. * *p* < 0.05, ** *p* < 0.01.

The average litter body weights were recorded daily from day 1 to day 8 after birth. The litters from sows supplemented with B.S-Dia showed a consistently higher average body weight across the entire observation period compared with the control group (Figure 1A). On day 8, the mean litter weight in the B.S-Dia group was 338 g higher than that in the control group, suggesting that maternal B.S-Dia supplementation improved early postnatal growth performance at the litter level. Furthermore, the jejunum morphology of the piglets in the different treatment groups was observed. For the piglets in the B.S-Dia treatment group, the jejunal villi were taller and crypts deeper compared to those of the control group (Figure 1B–D). Finally, the above results revealed that feeding B.S-Dia to sows enhanced piglet growth and intestinal epithelial development, which may contribute to improved mucosal function.

### 3.2. Difference in the Proliferation Status of the Jejunum After B.S-Dia Administration

**Figure 2:**
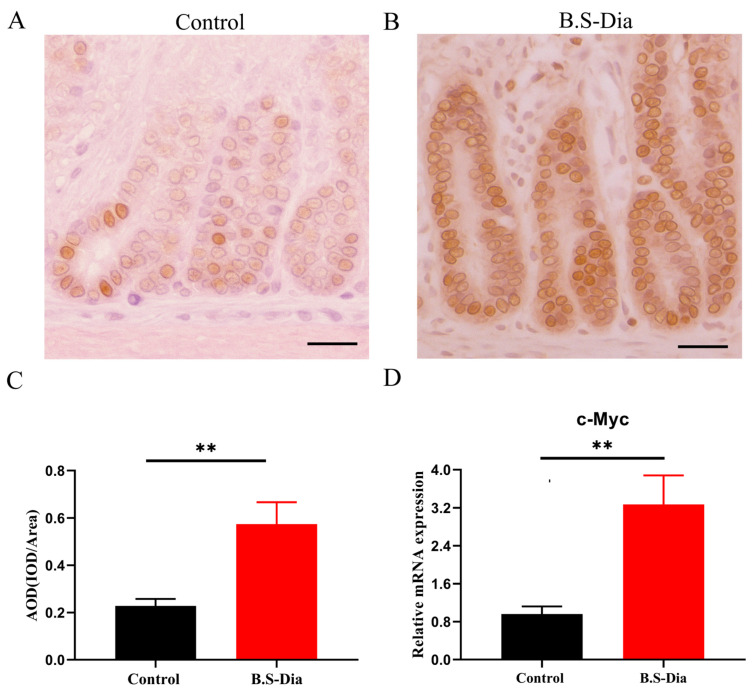
Jejunal cell proliferation activity was observed at the end of B.S-Dia administration. (**A**,**B**) PCNA^+^ cells in the crypt regions of the jejunum were visualized using immunohistochemical staining in both groups. Images were captured at original magnifications of ×400, scale bars = 25 μm. (**C**) AOD was quantified from ten tissue sections per group (*n* = 5). (**D**) mRNA expression of *c-Myc* genes detected using RT-qPCR (*n* = 5). Data are presented as mean ± SD, with significant difference determined using a *t*-test. ** *p* < 0.01.

The intestinal mucosa undergoes a continual process of proliferation, differentiation, and apoptosis. Gut epithelial cell proliferation is essential for the sustenance of gut cell homeostasis. To assess cellular proliferation in the jejunum, PCNA expression was evaluated via immunohistochemical staining and observed under a light microscope. Compared with the control group, the B.S-Dia-treated group exhibited a significantly increased proliferative index, as indicated by a higher average optical density (AOD) of PCNA-positive cells in the crypt regions (Figure 2A–C), accompanied by a slight upregulation in jejunal *c-Myc* mRNA expression (Figure 2D). Although increased *c-Myc* mRNA expression may be associated with enhanced cell proliferation, further mechanistic studies, such as on the knockdown or overexpression of *c-Myc*, are required to establish a causal relationship. In general, these data demonstrated that B.S-Dia enhanced intestinal epithelial proliferation, which promotes the maintenance of the gut mucosal barrier.

### 3.3. B.S-Dia Fed to Sows Improved Innate Immunity in Piglets

**Figure 3:**
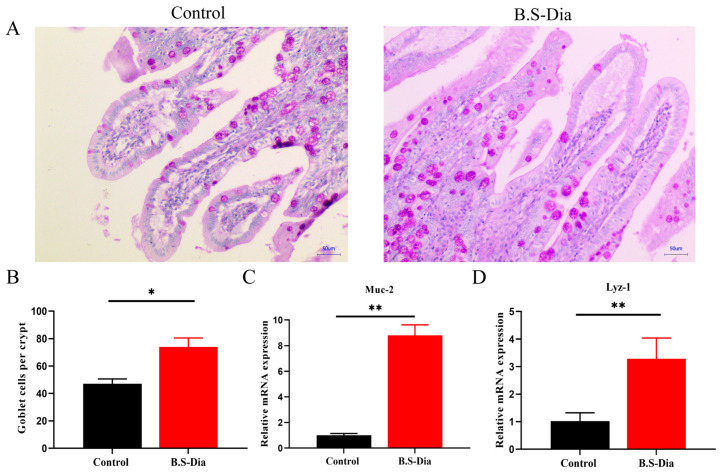
B.S-Dia supplementation in sows enhanced piglets’ innate immunity. (**A**) PAS staining revealed goblet cells in the jejunum at an original magnification of ×100, with a scale bar of 25 μm. (**B**) Data are expressed as mean ± SD; data was evaluation using a *t*-test. * *p* < 0.05. (**C**,**D**) RT-qPCR was used to detected the mRNA expression of *Muc2* and *Lyz-1* in the PBS and B.S-Dia groups, and data evaluation was conducted using a *t*-test. ** *p* < 0.01.

Multiple secretory cell types, including goblet and Paneth cells, are derived from intestinal stem cells. These two cell types contribute significantly to innate immunity by secreting antimicrobial peptides that protect against infections caused by pathogenic microorganisms [20,21]. In the present study, maternal B.S-Dia supplementation enhanced the innate immune defense of piglets, as evidenced by an increased number of goblet cells and the upregulated mRNA expression of antimicrobial peptides, including mucin (*Muc2*) and lysozyme (*Lyz-1*), in the jejunum (Figure 3A–D).

### 3.4. Activation of Mucosal Immunity in Piglets in the B.S-Dia Administration Group

**Figure 4:**
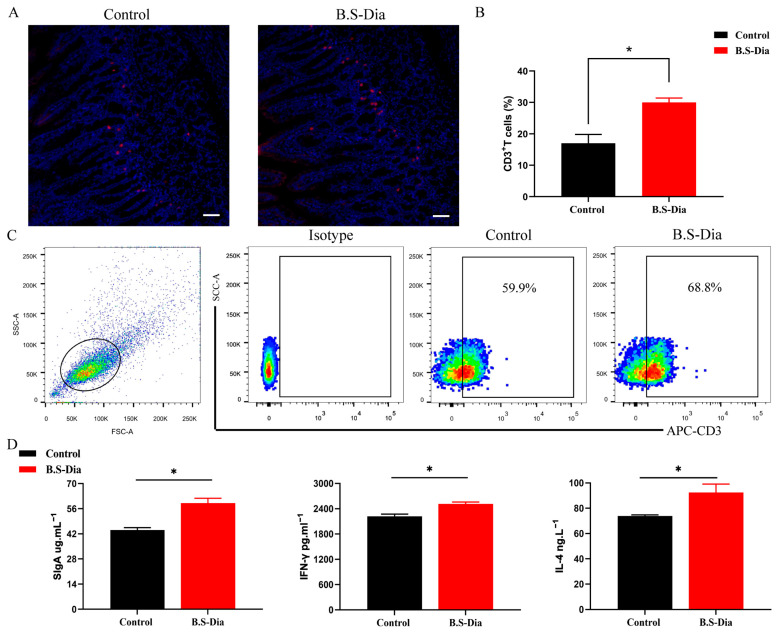
Impacts of mucosal immunity on the jejunum of piglets. (**A**,**B**) CD3^+^T cells in the jejunum were measured using immunofluorescence, original magnification ×100, with a scale bar of 50 μm. Data are expressed as mean ± SD; data evaluation was conducted using a *t*-test. * *p* < 0.05. (**C**) Flow cytometry was used to detect CD3^+^T lymphocytes in the blood. (**D**) SIgA, IFN-γ, and IL-4 concentrations in the gut rinse were assessed via ELISA kits. Statistical differences among the experimental groups were obtained via *t*-test, * *p* < 0.05.

A large number of immune cells are distributed in the intestinal mucosa, such as CD3^+^T cells, and these cells constitute a significant part of the intestinal mucosal immune system. In this study, the immunohistochemistry staining showed that the number of CD3^+^T cells in the jejunum markedly increased in the piglets from the B.S-Dia administration group (Figure 4A,B). Meanwhile, flow cytometry revealed that the number of CD3^+^T cells in the blood of piglets from the sows fed B.S-Dia also increased significantly (Figure 4C). Moreover, the ELISA results indicated that B.S-Dia administration significantly increased the concentration of SIgA in the jejunum (Figure 4D). SIgA can attach to the intestinal mucosa to maintain the integrity of the mucosa, and external pathogenic microorganisms discharge them. IL-4 promotes the differentiation of naive CD4^+^T cells into Th2 cells and induces immunoglobulin (Ig) class-switching to IgA and IgE [24]. IFN-γ, produced by activated Th1 cells, plays a key role in regulating the immune response and defending against viral and bacterial infections in piglets [25]. In this study, compared to the control group, the concentration of IL-4 and IFN-γ in the jejunum was slightly upregulated after B.S-Dia treatment (Figure 4D). These results suggested that B.S-Dia fed to sows enhanced the intestinal mucosal immune response.

## 4. Discussion

B.S is a feed additive commonly used in animal production, and has been widely applied to enhance the reproductive performance of sows and improve the intestinal mucosal immunity of weaned piglets. Previous studies have reported that dietary supplementation with B.S in sows significantly increases the total litter size, piglet birth weight, and concentrations of milk fat and milk protein [19,20,21]. Moreover, B.S supplementation in weaned piglets has been shown to improve the average daily gain and promote the development of the intestinal mucosal immune system [17,18]. However, there are few studies on improving the intestinal mucosal immune response of piglets by feeding them B.S. This may be due to the immaturity of the piglet immune system, which restricts the direct activation of mucosal immunity by B.S supplementation during the early postnatal period [26]. Therefore, maternal supplementation may be a promising strategy to improve the intestinal mucosal immunity of piglets. Sows have an epithelial–chorionic placenta [27]. Immunoglobulin and cytokines do not pass to the fetus through this type of placenta, so piglets only obtain immune substances through milk [27]. Compared with normal milk, sow colostrum contains more nutrients (milk fat and milk protein), cytokines, and immunoglobulins [28]. These nutrients and immune substances in the colostrum play an important role in promoting intestinal development and improving the intestinal mucosal immunity of piglets [29,30]. Our study was conducted to explore the impact of B.S-Dia fed to pregnant sows on piglets, specifically regarding piglets’ growth performance, intestinal morphology, cell proliferation, and the development of innate and mucosal immunity. The results of this study will provide a powerful basis for the application of B.S-Dia.

Due to the immaturity of the intestinal mucosal barrier in piglets, it serves as a potential entry point for pathogens, including *Escherichia coli* and porcine epidemic diarrhea virus [31,32]. It is necessary to stimulate intestinal development and enhance intestinal mucosal immunity to maintain piglet gut health. In this study, maternal B.S-Dia supplementation significantly improved piglet body weight and enhanced jejunal morphology, as evidenced by the increased villus height and crypt depth. These kinds of jejunal morphological enhancements lead to a larger absorptive surface area, which might also help clarify the weight gain seen in the piglets from the B.S-Dia group. Furthermore, the proliferative activity of intestinal epithelial cells following B.S-Dia treatment was assessed through PCNA staining and *c-Myc* mRNA expression analysis. The continuous proliferation of intestinal epithelial cells can mitigate the damage to the intestinal barrier caused by damaged cells, thus maintaining intestinal homeostasis [33,34]. This is beneficial for preventing pathogens from invading the piglets’ intestines.

Intestinal mucosal immunity consists of innate immunity and adaptive immunity. Innate immunity is the first line of defense and the basis for identifying and initiating inflammatory reactions against microorganisms [35]. Intestinal epithelial cells produce some innate immune substances when stimulated by the outside world, which is essential for preventing piglets from being infected by pathogenic microorganisms [33]. Derived from a shared stem cell progenitor, the gut epithelial layer consists of four principal cell subsets: absorptive enterocytes, goblet cells that produce mucus, enteroendocrine cells responsible for hormone release, and Paneth cells that secrete antimicrobial peptides [36]. In this study, the piglets within the B.S-Dia treatment group were able to increase the number of goblet cells. Interestingly, the goblet cells from the pigs in the group treated with B.S-Dia appeared larger compared to those from the control group. However, due to the limitations of our current study, we were unable to quantitatively assess the size differences of the goblet cells. We will certainly consider this as a future direction and plan to include this analysis in our subsequent research.

B.S-Dia supplementation also upregulated the mRNA expression of *Muc2* and *Lyz-1* in piglets, corresponding to the secretory functions of goblet and Paneth cells, respectively. Muc2 represents the major constituent of the mucus layer in the intestine and plays a key role in maintaining gut barrier integrity and creating a physical separation between the host epithelium and commensal bacteria. Enhanced Muc2 expression can limit bacterial adhesion and invasion, thereby shaping the composition and localization of the gut microbiota. Lyz-1 (lysozyme) is an antimicrobial peptide produced by Paneth cells that degrades bacterial cell walls, particularly the walls of Gram-positive bacteria. Its upregulation contributes to microbial homeostasis by directly suppressing pathogenic bacteria and supporting a balanced intestinal microbial community. Therefore, the observed increase in *Muc2* and *Lyz-1* expression may indicate that B.S-Dia supplementation supports gut health not only by enhancing mucosal defense, but also by modulating the intestinal microbiota through both physical and biochemical mechanisms.

In addition to the above two substances, the cytokine is also an important innate immune substance. IL-4 can promote the proliferation and type transformation of B lymphocytes, and IFN-γ affects the immune function in piglets and defends against viral and bacterial infections [24,25]. Our study revealed that the protein expression levels of IL-4 and IFN-γ were significantly upregulated in the B.S-Dia group. Additionally, both cytokines are essential for the differentiation of naive T cells, thereby modulating the adaptive immune response in piglet intestinal mucosa [29]. This was further supported by the increased CD3^+^T cell count following B.S-Dia administration. At the same time, the content of SIgA in the piglets’ intestines also increased significantly. SIgA attaches to the intestinal mucosa to maintain the integrity of the mucosa and effectively resists the invasion of pathogenic microorganisms that can damage the intestinal epithelial cells of piglets [37,38].

## 5. Conclusions

In conclusion, the treatment of sows with B.S-Dia effectively stimulates intestinal development, as evidenced by the increased villus height, crypt depth, and epithelial cell proliferation. B.S-Dia also increases the expression of innate immune substances, the concentration of SIgA, and the number of CD3^+^T cells, improving the intestinal mucosal immune function in piglets during early development. These findings indicate that B.S-Dia can not only improve piglet health by enhancing gut development and immune responses, but may also potentially improve the profitability of swine production by reducing diarrhea-related mortality and improving growth performance. Therefore, B.S-Dia supplementation may have significant applications in enhancing both the welfare of piglets and the productivity of swine farms.

## Data Availability

The raw data supporting the conclusions of this article will be made available by the authors upon request.

## Supplementary Materials

The following supporting information can be downloaded at: https://www.mdpi.com/article/10.3390/vetsci12050489/s1, Table S1: The primers used in this study
