# Supplementary material for: Bacillus subtilis Fed to Sows Promotes Intestinal Development and Regulates Mucosal Immunity in Offspring
Source: Vet Sci. 2025 May 18;12(5):489. doi: 10.3390/vetsci12050489 (PMC12116194; doi:10.3390/vetsci12050489)
Supplement: Supplementary file 1 [file vetsci-12-00489-s001.zip › vetsci-3614802-supplementary.pdf]

Table S1 The primers used in this study

Gene	Primer forward/Reverse	Length
Lyz-1-F	AACTGCTTTGGGTGTCTTGC	20bp
Lyz-1-R	GGTCTATGATCGGTGCGAGT	20bp
Muc2-F	GGGGTCCCCGTCTTCTTCAA	20bp
Muc2-R	GCGGTCCAGTCTGCTGTGTTG	21bp
c-Myc-F	CTCGGACTCTCTGCTCTCCT	20bp
c-Myc-R	TTGTTCCTCCTCAGAGTCGCT	21bp
GAPDH-F	GATGCCCTGGCCACAGAA	18bp
GAPDH-R	ACCCCTGCTCCCTCAACATC	20bp
